# Combined aerobic and resistance exercise training restores perivascular adipose tissue function in the thoracic aorta of rats with heart failure

**DOI:** 10.1042/CS20256965

**Published:** 2025-11-12

**Authors:** Milene Tavares Fontes, Daniela Esteves Ferreira dos Reis Costa, Patrizia Dardi, Suliana Mesquita Paula, Gisele Kruger Couto, Érique de Castro, Luciana Venturini Rossoni

**Affiliations:** 1 Department of Physiology and Biophysics, Institute of Biomedical Sciences, University of Sao Paulo, Sao Paulo, Brazil

**Keywords:** adiponectin, exercise training, heart failure, nitric oxide, noradrenaline, PVAT

## Abstract

Exercise training (ET) is increasingly recognized as a beneficial non-pharmacological intervention for cardiovascular diseases. Our previous results demonstrated that the thoracic perivascular adipose tissue (tPVAT) of heart failure (HF) rats underwent a phenotypic shift from brown to white adipose tissue, accompanied by impaired anticontractile function and oxidative stress. Thus, the present study aimed to investigate the effects of combined aerobic and resistance ET on the vasoactive properties of tPVAT in a HF rat model following myocardial infarction (MI). Wistar rats were subjected to either coronary artery ligation or sham operation (SO). Four weeks after surgery, the rats were divided into four groups: untrained (u) and exercise-trained (t) SO or HF. An 8-week ET program significantly improved running distance and maximum load lifting in the SO and HF groups, ameliorating tPVAT dysfunction and inducing browning only in the HF group. Additionally, ET enhanced the nitric oxide bioavailability, restored oxidative stress and pro-inflammatory cytokine levels (interleukin-6, tumor necrosis factor-α, and monocyte chemoattractant protein-1), and increased anti-inflammatory interleukin-10 levels in tPVAT. Furthermore, it increased noradrenaline (NE) content and β3-adrenoceptor (AR) gene expression in tPVAT, optimizing the NE/β3-AR/ adiponectin/AMP-activated protein kinase (AMPK)/endothelial nitric oxide synthase pathway locally. These findings highlight the potential of ET as a non-pharmacological approach to managing PVAT and vascular adjustments in HF.

## Introduction

Heart failure (HF) is a prevalent clinical syndrome characterized by structural and functional changes in the heart, resulting in increased cardiac filling pressures and decreased cardiac output. In addition to the heart, HF also affects other tissues, including the kidneys [1], skeletal muscle [2,3], conductance and resistance arteries [4–9], and veins [10]. Furthermore, we recently demonstrated that perivascular adipose tissue (PVAT) is also dysfunctional in HF after myocardial infarction (MI) [11].

Over the past 20 years, growing evidence has highlighted the physiological importance of PVAT in controlling vascular tone through its ability to reduce the contractile response of vascular smooth muscle cells—an effect known as the anticontractile effect of PVAT. This effect was first described by Soltis and Cassis, who demonstrated that PVAT reduces noradrenaline (NE)-induced contraction in rat aortas [12]. Functionally, the anticontractile effect of PVAT is observed in conductance and resistance arteries; however, the magnitude of the PVAT effect varies depending on the specific contractile agonist used [13]. This effect is associated with the action of adipokines and other vasoactive signaling molecules released from the PVAT in each vascular territory, such as nitric oxide (NO) and angiotensin 1–7 [14,15].

Importantly, impairment of the protective anticontractile effect of PVAT has been reported in several cardiometabolic diseases, including hypertension [16,17], diabetes [18], and obesity [19–21], and more recently in HF [11]. Our research group has demonstrated that the angiotensin-converting enzyme 1/angiotensin II/angiotensin II type 1 and type 2 receptor axes are crucial to the dysfunction of thoracic aorta PVAT (tPVAT) in HF rats post-MI [11]. Dysfunctional tPVAT exhibits lower NO bioavailability, oxidative stress, and whitening—changes that suggest impaired secretory function, which underlies the loss of its anticontractile properties in HF [11].

The thoracic aorta acts as an elastic buffer at the beginning of the systemic circulation, playing a central role in dampening the pulsatile output and ensuring continuous blood flow to the periphery. Its mechanical features (elasticity and stiffness) are key determinants of the Windkessel function, thereby influencing left ventricle (LV) afterload and coronary circulation [22,23]. In HF, reduced aortic compliance increases the effort required for the LV to eject blood [24]. Vascular dysfunction, aortic remodeling, and stiffness, which are hallmarks of HF, not only modify cardiac afterload but also provide prognostic information [24–27]. Thus, it is crucial to comprehend the active and passive properties of the thoracic aorta in HF, as changes in this vessel directly affect the LV and the microcirculation, thereby inducing a progressive and pathological cycle.

Exercise training (ET) is widely recognized as a crucial therapeutic approach for preventing and treating cardiovascular diseases non-pharmacologically, including HF [3,20,28–30]. Our research group has demonstrated that aerobic ET can reverse coronary endothelial dysfunction, loss of myogenic response, and peripheral edema associated with HF [7,8]. Moreover, the beneficial effects of aerobic ET extend to PVAT, restoring its anticontractile effect in conditions such as obesity [20,31–33]. Muscle mass loss in HF is associated with an increased risk of mortality in hospitalized patients; therefore, resistance training is an important strategy for enhancing or maintaining muscle mass and strength [34,35]. Thus, the combination of aerobic and resistance ET is indicated in guidelines for both healthy individuals and those with cardiovascular conditions [33–38]. This combined approach enhances the overall therapeutic benefits due to the distinct stimuli promoted by each exercise type [38,39]. However, whether combined aerobic and resistance ET can also restore the impaired anticontractile function of tPVAT in HF post-MI remains unclear. Therefore, we hypothesize that combined aerobic and resistance ET will effectively reverse the impaired anticontractile effect of tPVAT in HF. Thus, we investigated the effects of this combined exercise approach on tPVAT function in HF rats, as well as the underlying mechanisms involved.

## Material and methods

### Experimental model of HF

All experimental procedures were approved by the Ethics Committee on Animal Use at the Institute of Biomedical Sciences of the University of Sao Paulo (No. 53, Sheet 19, Book 3) and were conducted in accordance with the Brazilian Council for Animal Research and the National Institute of Health Guide for the Care and Use of Laboratory Animals. Male Wistar rats (250 ± 20 g) were obtained from the Animal Facility of the Institute of Biomedical Sciences of the University of Sao Paulo and were kept in the Animal Facility of the Department of Physiology and Biophysics under controlled conditions of temperature and humidity, with a 12:12 h light-dark cycle and free access to water and food. Eight-week-old animals were anesthetized using ketamine–xylazine (90 and 10 mg/kg, i.p., respectively; Sespo Indústria e Comércio, Paulínia, SP, Brazil) and subjected to MI by permanent occlusion of the left coronary artery or to a sham operation (SO), as previously described [6].

Four weeks after MI, blood samples were collected from the retro-orbital plexus of the rats using a heparinized capillary tube. The animals were anesthetized (3–4% isoflurane in oxygen) to minimize discomfort during the procedure. The capillary tube was gently inserted into the medial canthus of the eye, and a 200-µL blood sample was withdrawn. The brain natriuretic peptide (BNP) plasma levels were assessed using an ELISA kit (cat. no. ab108815, Abcam, San Francisco, CA, USA). As previously reported, BNP levels greater than 0.3 ng/mL served as the inclusion criterion for HF animals in the study [8]. In line with this parameter, plasma levels of BNP were significantly increased in HF rats compared with SO rats (SO: 0.12  ±  0.01 (*n* = 26) vs. HF: 0.68  ± 0.1 ng/mL (*n* = 25); *P*<0.05, unpaired t-test).

### Exercise training

Afterward, ET was performed on an ergometric treadmill (aerobic training) and a ladder adapted for rats (resistance training) on alternate days (combined training), 5 days a week, for 1 h per day, over an 8-week period. The rats were subdivided into the following four groups: (1) untrained SO (uSO), (2) trained SO (tSO), (3) untrained HF (uHF), and (4) trained HF (tHF).

#### Treadmill exercise training protocol

Rats from both sedentary and trained groups underwent a 1-week acclimatization period on the ergometric treadmill (15–20 min at a speed of 0.3–0.6 km/h, 0% inclination, daily). Subsequently, the rats were submitted to the first maximum effort capacity test, starting at 0.3 km/h with increments of 0.3 km/h every 3 min until exhaustion, to determine the maximum speed achieved. The exercise-trained rats (tSO and tHF groups) underwent an 8-week training period on a treadmill (at 50 to 60% of the average maximal speed from the pretraining exercise capacity test, with 0% inclination) [7,8]. The sedentary rats (uSO and uHF groups) were subjected to a short period of exercise, once a week, for 10 min at a speed of 0.3 km/h [7,8]. The maximum effort capacity test was repeated in weeks 4 and 8 of the protocol to adjust exercise intensity and evaluate the increase in aerobic capacity between sedentary and trained rats.

#### Ladder exercise training protocol

Similarly, rats from both the sedentary and trained groups were subjected to a 1-week acclimatization period on a ladder (1.1 m high × 0.18 m wide, with a 2-cm grid and an 80° incline). At the end of the acclimatization period, the rats were submitted to the first maximum load test, which consisted of an initial load of 75% of body weight attached to the animal’s tail with adhesive tape, with increments of 25% of body weight in subsequent climbs until exhaustion [40]. The exercise-trained rats (tSO and tHF groups) were submitted to an 8-week training period of moderate intensity (40–60% of the maximal load), as recommended for HF patients [35,41]. The sedentary rats (uSO and uHF groups) were placed on the ladder once a week, without overload, to maintain familiarity with climbing a ladder. The maximum load capacity test was repeated in weeks 4 and 8 of the protocol to adjust the exercise intensity and evaluate the force capacity among groups.

### Hemodynamic and morphometric evaluations

LV hemodynamic parameters were measured in anesthetized animals (urethane, 1.2 g/kg, i.p.; Sigma-Aldrich, St. Louis, MO, USA), as previously described [6], either 48 h after the final 8-week training session or at 12 weeks post-MI. Then, the rats were killed by exsanguination, and the heart, lungs, tibia, and thoracic aorta (with PVAT) were carefully removed. The right ventricle (RV) and LV hypertrophy indexes, as well as the degree of pulmonary congestion, were inferred from tissue weight normalized to tibia length. The scar tissue post-MI was evaluated by planimetry and expressed as a percentage of the total LV area (including the septum). Only rats that presented infarcted areas covering at least 30% of the LV surface were included in the study [6–8,11]. Thus, the infarcted area of LV at 12 weeks post-MI was similar between the tHF and uHF groups (uHF: 34.95 ± 0.97 (*n* = 22) vs. tHF: 34.07 ± 0.74% (*n* = 20); *P*>0.05, unpaired t-test).

### Vascular reactivity studies

As previously published, the thoracic aorta was sectioned into 3-mm rings with or without tPVAT (mechanically denuded) [11]. Afterward, phenylephrine-induced concentration-response curves (0.1 μmol/L–300 μmol/L) were assessed in aortic rings that had been previously incubated, or not, with the non-selective nitric oxide synthase (NOS) inhibitor L-NAME (100 μmol/L; Sigma-Aldrich) for 30 min. The phenylephrine-induced contractile response was measured in mN using a data acquisition system (MP 100, Biopac Systems Inc., Goleta, CA, USA) and normalized to the ring length (mN/mm).

### Gene expression analysis

According to the manufacturer’s instructions, total RNA was isolated from the tPVAT using Trizol reagent (Invitrogen, Carlsbad, CA, USA). cDNA was synthesized using the qPCR-SuperMix-UDG Kit (Invitrogen) from 1 μg of RNA in a total volume of 20 μL with oligo (dT) primers (Invitrogen). Quantitative RT-PCR was performed using a thermocycler (Corbett Research, Sydney, Australia) with SYBR Green PCR (Invitrogen). cDNA served as the template for PCR with the following specific primers (Table 1).

**Table 1:** Sequence of primers used in the study

Genes	Sequence of primers
PRDM-16	Sense: 5′TGATGGCCGCTTGGAAGA 3′ Anti sense: 3′TCACTGCCATCCGACATGTC 5′
EPSTI-1	Sense: 5′TGACGGCTGGGGTATATGAGA 3′ Anti sense: 3′AGTGGGTGGGCAGTTGAAAT 5′
TCF-21	Sense: 5′TCCAAGCTGGACACTCTCAG 3′ Anti sense: 3′ TAAAGGGCCAAGTCAGGTTGA 5′
UCP1	Sense: 5′ATCTTCTCAGCCGGCGTTTC 3′ Anti sense: 3′CCTTGGATCTGAAGGCGGAC 5′
mtTFA	Sense: 5′ATTCCGAATTGTTTTTCCAGCA 3′ Anti sense: 3′TCTGAAAGTTTTGCATCTGGGT 5′
eNOS	Sense: 5′GGATCCAGTGGGGGAAACTG 3′ Anti sense: 3′TGGCTGAACGAAGATTGCCT 5′
IL-6	Sense: 5′CATTCTGTCTCGAGCCCACC 3′ Anti sense: 3′GCTGGAAGTCTCTTGCGGAG 5′
TNF-α	Sense: 5′GTGATCGGTCCCAACAAGGA 3′ Anti sense: 3′CTTGGTGGTTTGCTACGACG 5′
MCP-1	Sense: 5′TGTCTCAGCCAGATGCAGTT 3′ Anti sense: 3′CAGCCGACTCATTGGGATCA 5′
IL-10	Sense: 5′ACGCTGTCATCGATTTCTCCC 3′ Anti sense: 3′GTCACGTAGGCTTCTATGCAGT 5′
β3-AR	Sense: 5′TAGCAAGGAGCCTGACTTCTG 3′ Anti sense: 3′TTGGTTCTGGAGAGTTGCGG 5′
HPRT1	Sense: 5′ACAGGCCAGACTTTGTTGGA 3′ Anti sense: 3′TGGCTTTTCCACTTTCGCTG 5′

As previously described, samples were run in duplicate [11]. Gene expression of brown [PR-domain containing 16 (PRDM-16)], beige [epithelial stromal interaction 1 (EPSTI-1)], and white [transcription factor-21 (TCF-21)] adipose tissue markers; the uncoupling protein 1 (UCP1) and mitochondrial transcription factor A (mtTFA); endothelial nitric oxide synthase (eNOS); pro-inflammatory markers [interleukin-6 (IL-6), tumor necrosis factor-α (TNF-α), and monocyte chemoattractant protein-1 (MCP-1)]; anti-inflammatory marker [interleukin-10 (IL-10)]; and the β3-adrenoceptor (AR) were evaluated. Analysis of the gene of interest was normalized to the mRNA levels of hypoxanthine–guanine phosphoribosyl transferase 1 (HPRT-1). Data were expressed as the fold induction relative to the control.

### Adiponectin, angiotensin II, and noradrenaline enzyme immunoassay

Adiponectin, angiotensin II, and NE concentration were measured using commercial ELISA kits [Rat Total Adiponectin/Acrp30 Immunoassay, Cat. RRP300, R&D Systems, Minneapolis, MN, USA; Angiotensin-II (human, rat, mouse) Extraction Free EIA Kit Protocol, Cat. EKE-002–12, Phoenix Pharmaceuticals, CA, USA; and Noradrenaline Research Elisa, Cat. BA E-5200R, Labor Diagnostika Nord, GmbH and Co. KG, Nordhorn, Germany, respectively].

For adiponectin analysis, samples of tPVAT were homogenized in PBS (30 mg of tissue per 300 μL of PBS) and centrifuged (2,300 g for 20 min at 4°C). The protein content in the supernatant was quantified by spectrophotometry using the BCA method (Thermo Scientific Pierce, IL, USA). Then, all samples were diluted in assay diluent (Calibrator Diluent RD5-26, Rat Total Adiponectin/Acrp30 Immunoassay) at a final concentration of 10 ng/μL. Subsequently, adiponectin was quantified according to the manufacturer’s instructions, and the results were expressed as ng of adiponectin per μg of protein.

Segments of tPVAT were also homogenized with acid-ethanol solution [87.5% ethanol and 12.5% HCl (2 mol/L)] to measure angiotensin II and NE concentrations. Upon homogenization, a mixture of five parts acid-ethanol solution and one part tissue homogenate was incubated for 30 min at 20°C and centrifuged (3,000 g for 30 min at 4°C). The resulting supernatant was transferred into new tubes and subjected to evaporation using a centrifugal concentrator (CentriVap Concentrator, Labconco, MO, USA) until complete dryness was achieved. This dried extract was reconstituted with 1× assay buffer (Angiotensin-II (human, rat, mouse) Extraction Free EIA Kit Protocol), resulting in a fourfold concentration enhancement. The BCA method (Thermo Scientific Pierce) was used to quantify proteins by spectrophotometry. For the quantification of angiotensin-II content, 10 µg of tPVAT homogenate was used, and for NE content, 10 µL of the homogenate or rats’ plasma was employed, following the manufacturer’s guidelines for each assay. The tPVAT angiotensin II and NE levels were expressed as ρg/mL/µg of protein and ng/mL/µg of protein, respectively. Plasma NE analysis was expressed as ng/mL.

### Western blotting analysis

Total protein extracts were obtained from tPVAT. Tissues were homogenized in cold RIPA lysis buffer (Merck Millipore, Billerica, MA, USA) containing phenylmethylsulfonyl fluoride (1 mM), sodium orthovanadate (Na3VO4, 1 mM), and a protease inhibitor cocktail (2 μL/mL; Sigma-Aldrich). Protein extracts (75 μg) were separated by SDS–PAGE and transferred overnight at 4°C to polyvinylidene fluoride (PVDF) membranes (GE HealthCare, Little Chalfont, Buckinghamshire, UK) using a Mini Trans-Blot Cell system (Bio-Rad, Hercules, CA, USA) with transfer buffer (25 mM Tris, 190 mM glycine, 20% methanol, and 0.05% SDS). Membranes were blocked for 90 min at room temperature with 5% albumin in Tris buffer (10 mM Tris, 100 mM NaCl, and 0.1% Tween 20) and then incubated overnight at 4°C with the primary antibodies: anti-phospho-AMPKα (Thr172) (1:1,000; Cell Signaling Technology, Danvers, MA, USA), anti-AMPKα1/2 (1:1,000; Santa Cruz Biotechnology, Dallas, TX, USA), and anti-tyrosine hydroxylase (1:1,000; Immunostar, Hudson, WI, USA). After washing, membranes were incubated for 90 min with a peroxidase-conjugated IgG antibody specific to the primary antibody used. Protein expression was detected using Pierce ECL Western Blotting Substrate (Thermo Scientific, Rockford, IL, USA), and the membranes were subjected to autoradiography using Amersham Hyperfilm ECL (GE Healthcare). The blots were digitized, and the intensity was quantified using ImageJ 1.46 p software (National Institutes of Health, Bethesda, MD, USA). Ponceau staining was employed to normalize the expression of the evaluated proteins in each sample, as previously published [42].

### Reactive oxygen species evaluation

Aortic rings with tPVAT (3 mm) from all groups were incubated for 30 min in oxygenated Krebs–Henseleit solution (pH 7.4, 37°C) with dihydroethidium (DHE, 2 μmol/L; Life Technologies, Carlsbad, CA, USA) in a dark chamber for *in situ* reactive oxygen species (ROS) measurement, as previously described [11,42]. Subsequently, these rings were fixed in 4% paraformaldehyde solution for 4 h and then embedded in a freezing medium (Tissue-Tek, Sakura Finetek, Torrance, CA, USA). Transverse sections (10 μm thick) of arteries with PVAT were equilibrated in phosphate buffer (0.1 mol/L, pH 7.4) for 10 min at 37°C, and images (20× objective) were taken using an optical microscope (Eclipse 80i, Nikon) equipped with a rhodamine filter. The images were analyzed using ImageJ software. The ROS production was evaluated and expressed as the integrated density of DHE fluorescence.

### Statistical analysis

Values are presented as mean ± SEM. The ROUT test was used to identify outliers, and the Shapiro–Wilk test was employed to assess the normality of the data. Student’s t-test or one- and two-way analysis of variance (ANOVA) was used as appropriate, followed by the Bonferroni or Tukey post-hoc test (GraphPad Prism Software, San Diego, CA, USA). A *P*<0.05 was considered statistically significant.

## Results

As previously published [7,8], uHF rats exhibit a lower maximal running distance than the uSO group (Table 2). However, no significant changes were observed in the maximal load lifted on the ladder (Table 2). After an 8-week ET protocol, both trained groups demonstrated a longer running distance on the treadmill and a higher maximum load lifted on the ladder compared with their respective untrained groups (Table 2). These results confirm the effectiveness of training in enhancing physical activity in SO rats and restoring it in HF rats.

**Table 2:** Hemodynamic and morphometric parameters, as well as performance testing (maximum distances and loads), were evaluated among groups after 8 weeks of either a sedentary lifestyle or combined training

	uSO	tSO	uHF	tHF
n	28	37	22	20
**Hemodynamic values**				
LVSP (mmHg)	140 ± 1.90	143 ± 1.94	125 ± 2.10*	122 ± 2.61*
LVeDP (mmHg)	6.13 ± 0.37	5.88 ± 0.22	18.28 ± 2.36*	11.95 ± 1.14*^#^
+ dP/dt (mmHg/s)	7294 ± 254.36	7586 ± 268.71	5397 ± 267.00*	5480 ± 301.38*
− dP/dt (mmHg/s)	−4645 ± 148.98	−4744 ± 135.84	−3803 ± 183.24*	−3994 ± 201.78*
HR (beats/min)	346 ± 7.88	360 ± 6.24	347 ± 6.76	338 ± 7.07
**Morphometric values**				
Body weight (g)	477 ± 10.61	471 ± 9.54	458 ± 25.11	459 ± 11.80
Tibia length (mm)	41.89 ± 0.20	41.68 ± 0.17	41.73 ± 0.40	41.74 ± 0.27
LVW/Tibia (mg/mm)	19.24 ± 0.40	19.51 ± 0.40	19.55 ± 0.61	19.61 ± 0.41
RVW/Tibia (mg/mm)	5.34 ± 0.14	5.31 ± 0.16	12.69 ± 0.83*	11.26 ± 0.63*
Lung/Tibia (mg/mm)	46.06 ± 1.7	47.26 ± 1.86	90.20 ± 4.48*	85.72 ± 3.55*
**Performance testing**				
Running distance (m)	135 ± 7.31	352 ± 28.35*	96 ± 7.62*	246 ± 23.94*^#^
Maximal load (g)	563 ± 11.12	767 ± 13.13*	482 ± 14.62	660 ± 13.97*^#^

Values are presented as the mean ± SEM. n, number of animals; uSO: untrained sham-operated; tSO: trained sham-operated; uHF: untrained heart failure; tHF: trained heart failure; LV, left ventricle; LVSP, left ventricle systolic pressure; LVeDP, left ventricle end-diastolic pressure; +dP/d*t*, first-time positive derivative; −dP/d*t*, first-time negative derivative; HR, heart rate; LVW/Tibia, ratio of left ventricle weight to tibia length; RVW/Tibia, ratio of right ventricle weight to tibia length; Lung/Tibia, ratio of lung weight to tibia length. Significance was assessed using two-way ANOVA. *P<0.05 vs. uSO and ^#^ *P*<0.05 vs. uHF.

In addition, the uHF rats presented significant cardiac dysfunction compared with uSO, as demonstrated by the reduction in LV systolic pressure and pressure to time derivatives (+dP/dt, −dP/dt), as well as the enhancement in the LV end-diastolic pressure (LVeDP) (Table 2). Combined ET was able to reduce the LVeDP level only in HF rats, without changes in the other hemodynamic variables analyzed (Table 2). The RV and lung weight to tibia length ratios were higher in uHF rats than in uSO rats, and training did not alter these parameters (Table 2). The other morphometric parameters did not change significantly in either HF or with combined training (Table 2).

### Combined exercise training improves the anticontractile profile and induces browning in the tPVAT of HF post-MI rats

**Figure 1:**
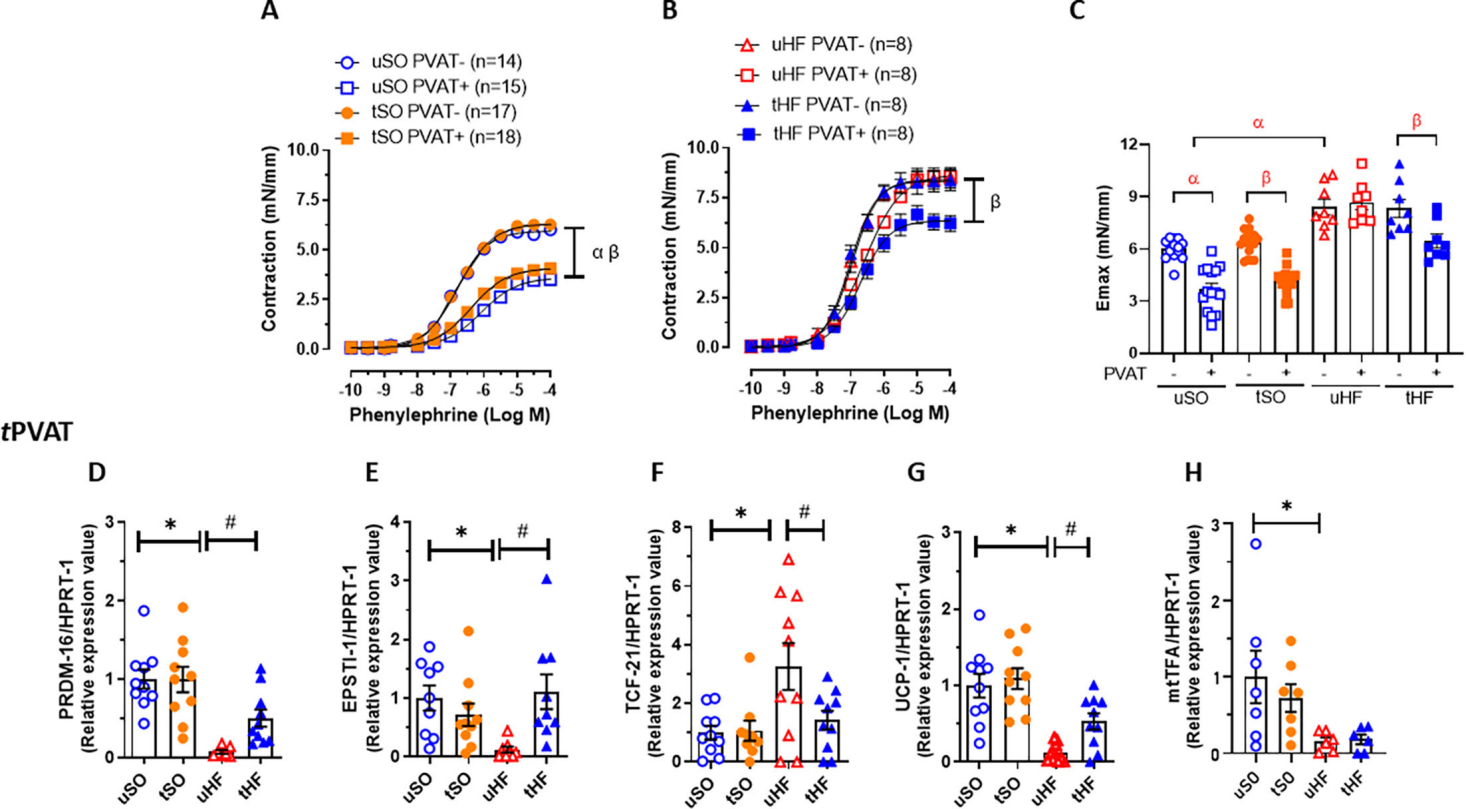
Exercise training improves the anticontractile effect of tPVAT and induces browning in heart failure rats. Concentration-response curves to phenylephrine in thoracic aortic rings with (+) and without (−) PVAT from (**A**) untrained sham-operated (uSO) and training sham-operated (tSO) rats; (**B**) untrained heart failure (uHF) and training heart failure (tHF) rats; and (**C**) maximum effect values (Emax) obtained in each condition and group. Gene expression of (**D**) brown (PRDM-16), (**E**) beige (EPSTI-1), and (**F**) white (TCF-21) adipose tissue markers, and (**G**) UCP1 and (**H**) mtTFA mitochondrial metabolic and biogenic markers in tPVAT from the studied groups. All results are expressed as mean ± SEM. Gene expression is presented as the fold of induction to the uSO group. A two-way analysis of variance (ANOVA) followed by the Tukey post-hoc test was used to compare the results. *P<0.05 vs. uSO; ^#^ *P*<0.05 vs. uHF; ^α^ *P*<0.05 vs. untrained PVAT−; *^β^P*<0.05 vs. trained PVAT−. HPRT-1, hypoxanthine–guanine phosphoribosyl transferase 1.

The presence of tPVAT significantly reduced the contraction induced by phenylephrine in the aorta of the uSO group, and ET did not alter the contractile response in either aortic ring with (+) or without (−) PVAT (Figure 1A and C). As previously demonstrated by our group [11], uHF exhibited an increase in phenylephrine-induced contraction in both the PVAT+ and PVAT− aortic rings compared with uSO rats (Figure 1B and C). However, for the first time, our results showed that the anticontractile effect of tPVAT was restored after the 8-week ET program in HF rats, whereas no changes were observed in the PVAT− aortic rings (Figure 1B and C). It is essential to note that, although ET enhances the anticontractile effect of tPVAT in the HF group, the magnitude of this effect does not return to the uSO level (compare Figure 1A and B).

We have also previously demonstrated that tPVAT of HF rats significantly changed the adipocyte phenotype, despite no change in its amount [11]. Therefore, we evaluated whether combined ET could restore the adipocyte phenotype of PVAT. tPVAT of uHF rats presented a reduction in the gene expression of brown (PRDM-16, Figure 1D) and beige (EPSTI-1, Figure 1E) adipose tissue markers, as well as in the mitochondrial marker UCP1 (Figure 1G) and the mitochondrial transcription factor mtTFA (Figure 1H), when compared with uSO. Conversely, increases in the expression of all these genes were found in the tHF group, except for mtTFA, which remained reduced despite the training protocol. On the other hand, the gene expression of the white adipose tissue marker TCF-21 (Figure 1F) was increased in uHF tPVAT compared with uSO, and ET was able to reduce TCF-21 expression in the tPVAT of tHF. Moreover, ET did not alter these markers in the tPVAT of the SO group compared with the uSO group (Figure 1D–H).

Altogether, these results demonstrate for the first time that the combined aerobic and resistance ET is a non-pharmacological tool that induces browning and restores the anticontractile effect of tPVAT in HF post-MI rats, thereby improving vascular tone regulation. However, it did not alter the vasoactive profile or the gene expression of adipocyte markers in the tSO group. Hereafter, we focused our study only on uSO, uHF, and tHF rats.

### Combined exercise training restores eNOS activation on tPVAT of HF post-MI rats

**Figure 2:**
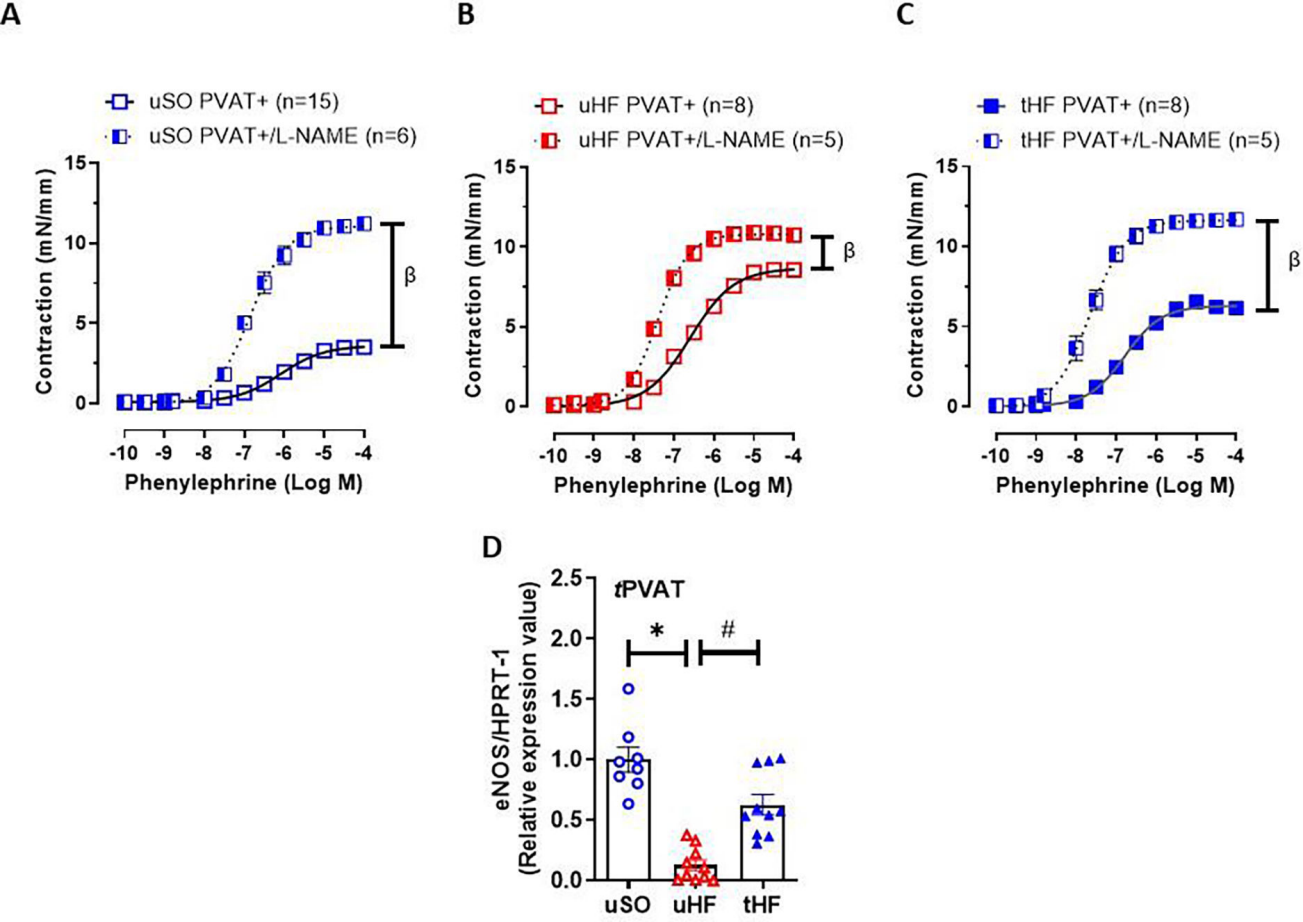
Exercise training enhances NO bioavailability and eNOS gene expression in the tPVAT of heart failure rats. Concentration-response curves to phenylephrine in thoracic aortic rings PVAT+ from (**A**) untrained sham-operated (uSO); (**B**) untrained heart failure (uHF); and (**C**) training heart failure (tHF) rats, previously incubated, or not, with L-NAME (100 μmol/L) for 30 min. (**D**) Gene expression of eNOS was detected in tPVAT from the studied groups. All results are expressed as mean ± SEM. Gene expression is presented as the fold of induction to the uSO group. A two- or one-way analysis of variance (ANOVA) followed by the Tukey post-hoc test was used to compare the results. *P<0.05 vs. uSO; ^#^ *P*<0.05 vs. uHF; *^β^P*<0.05 vs. PVAT+.

The importance of NO for physiological tPVAT function, as well as its role in HF dysfunction, is already well established [11,42], as is the ability of training to modulate NO synthesis and bioavailability [7,43,44]. Therefore, we investigated the effect of ET on NOS activation in aortic rings with intact PVAT. Inhibition of NOS activity with L-NAME increased the contraction induced by phenylephrine in PVAT+ aortic rings of uSO rats, whereas a minor increase was observed in the uHF group compared with the uSO group (compare Figure 2A and B). Remarkably, ET in HF rats restored the effect of L-NAME in PVAT+ aortic rings to that of the uSO group (compare Figure 2A and C). In line with this result, gene expression of eNOS was lower in tPVAT of uHF rats than in uSO, whilst ET enhanced this gene expression to uSO levels (Figure 2D). Together, these findings suggest that the beneficial effects of the combined aerobic and resistance ET on tPVAT function are mediated by the activation of eNOS.

### Combined exercise training has an antioxidant and anti-inflammatory effect in the tPVAT of HF post-MI rats

**Figure 3:**
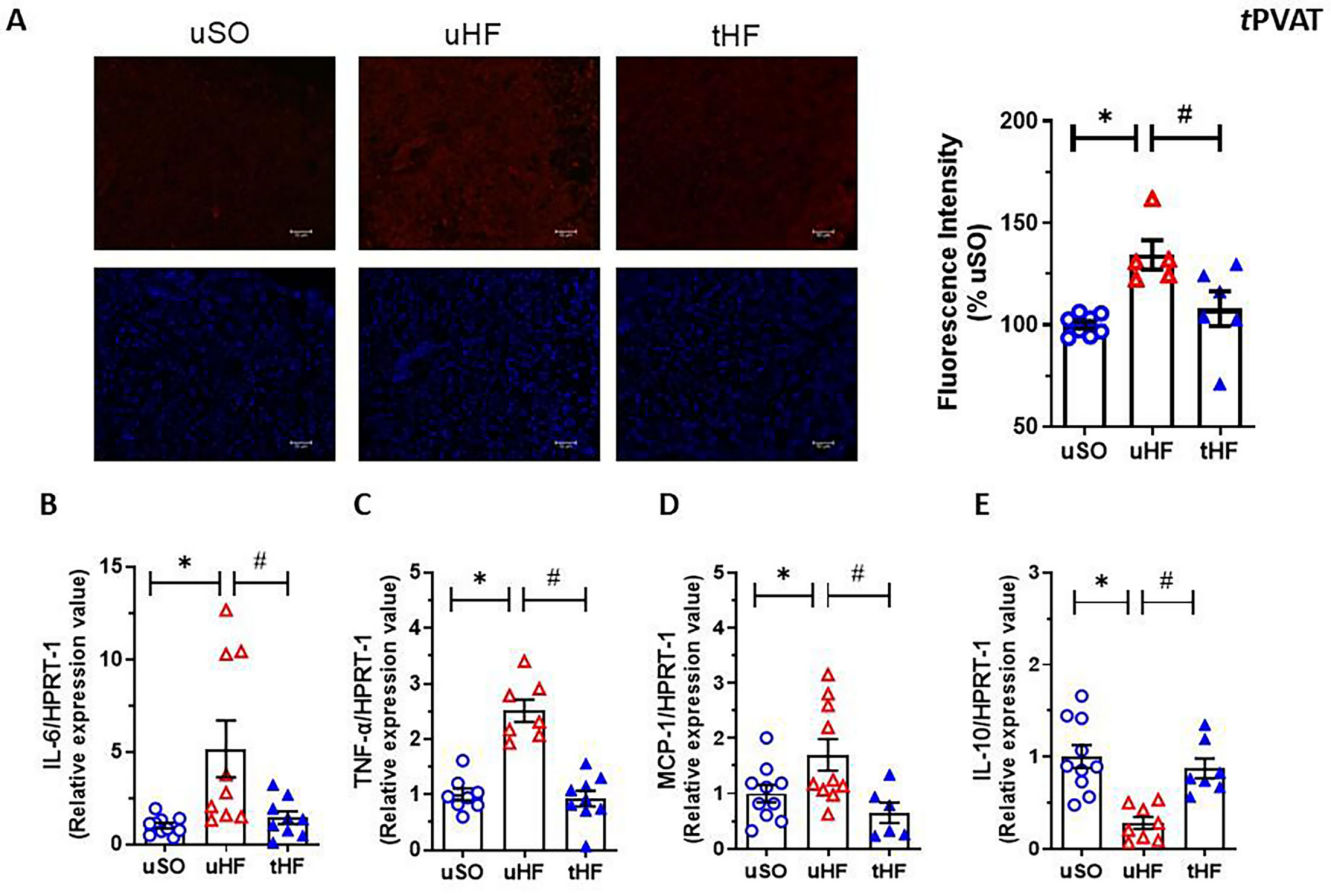
Exercise training improves the antioxidant and anti-inflammatory profile of tPVAT of heart failure rats. (**A**) Representative laser-scanning fluorescent micrographs of tPVAT from (**A**) untrained sham-operated (uSO); (**B**) untrained heart failure (uHF); and (**C**) training heart failure (tHF) rats, preloaded with dihydroethidine (DHE) for ROS detection, or 4′,6-diamidino-2-phenylindole (DAPI) for nuclei detection. The histogram showed the mean of fluorescence intensity (% emission/nucleus to the uSO group) of DHE dye in slices of tPVAT from each group. Gene expression of (**B**) IL-6; (**C**) TNF-α; (**D**) MCP-1; and (**E**) IL-10 detected in tPVAT from studied groups. All results are expressed as mean ± SEM. Gene expression is presented as the fold of induction to the uSO group. One-way analysis of variance (ANOVA) followed by the Tukey post-hoc test was used to compare the results. *P<0.05 vs. uSO; ^#^ *P*<0.05 vs. uHF.

As evidenced in Figure 3A (typical image and bar graph), ROS production was higher in the tPVAT of the uHF group compared with the uSO group, as demonstrated by the increase in DHE fluorescence intensity. ET restored the DHE fluorescence intensity in the tPVAT of the tHF rats to the level found in the uSO group (Figure 3A).

In addition, ET was able to attenuate the increase in gene expression of the pro-inflammatory markers IL-6 (Figure 3B), TNF-α (Figure 3C), and MCP-1 (Figure 3D) observed in tPVAT of uHF rats to the levels of uSO. On the other hand, gene expression of IL-10, an anti-inflammatory cytokine, was significantly reduced in the tPVAT of the uHF group compared with the uSO group, and ET was able to restore it to the level found in the uSO group (Figure 3E).

These findings suggest that the combined aerobic and resistance ET has effective antioxidant and anti-inflammatory effects on the tPVAT of HF post-MI rats.

### Combined exercise training restores noradrenaline content but does not change angiotensin II levels on tPVAT of HF post-MI rats

**Figure 4:**
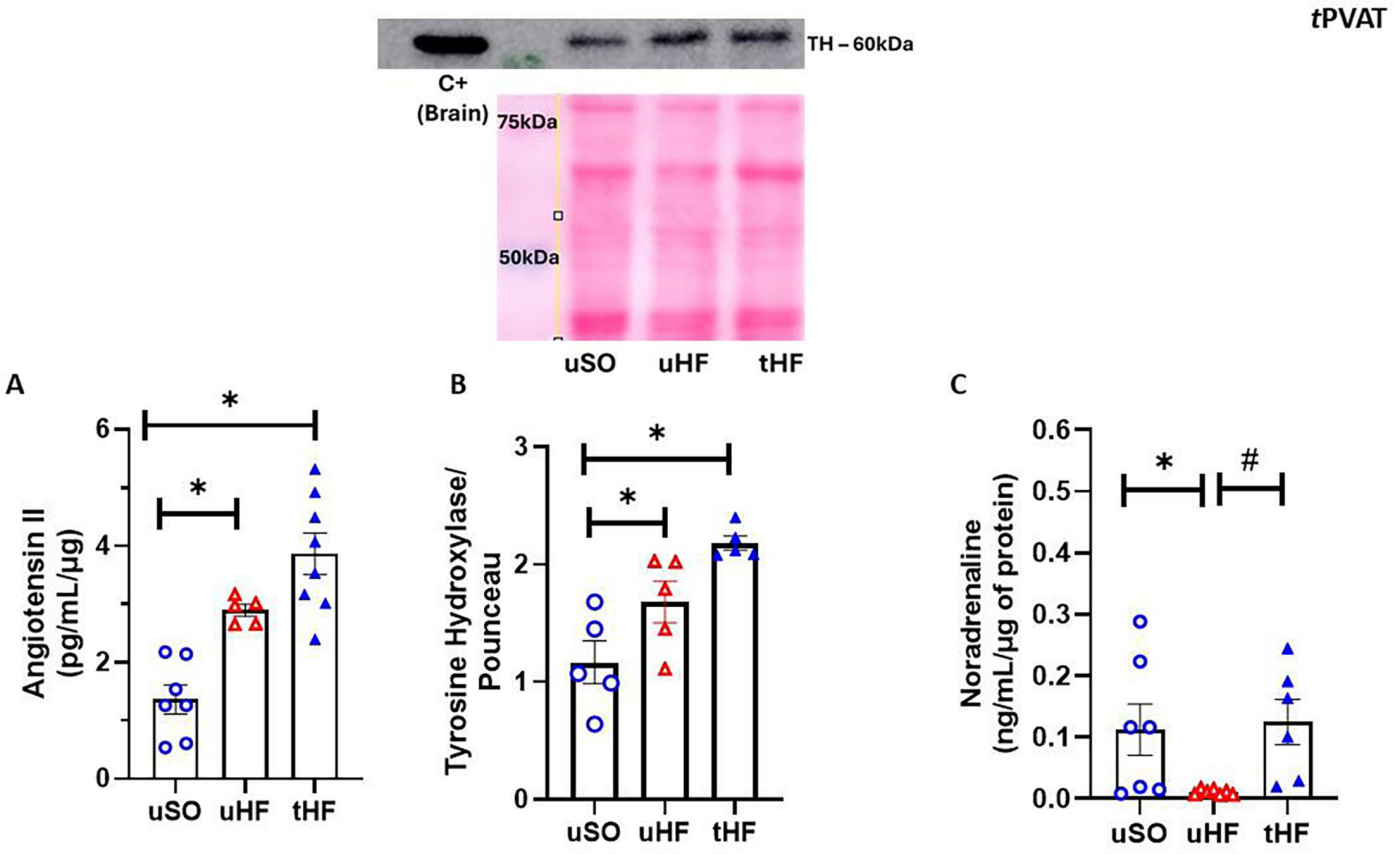
The effect of exercise training on tPVAT of heart failure rats involved the noradrenaline pathway but not the angiotensin II system. (**A**) Angiotensin II levels; (**B**) protein expression of tyrosine hydroxylase; and (**C**) noradrenaline levels detected in tPVAT from untrained sham-operated (uSO); untrained heart failure (uHF); and training heart failure (tHF) rats. All results are expressed as mean ± SEM. Ponceau staining was used to normalize the expression of the evaluated protein. One-way analysis of variance (ANOVA) followed by the Tukey post-hoc test was used to compare the results. *P<0.05 vs. uSO; ^#^ *P*<0.05 vs. uHF.

We previously demonstrated that tPVAT from HF rats presents higher levels of angiotensin II, which was associated with the loss of the anticontractile effect of this tissue [11]. Angiotensin II has pro-oxidant and pro-inflammatory effects, reducing NO bioavailability, which may contribute to vascular dysfunction [15,45]. Additionally, it is well established that ET modulates the renin-angiotensin system (RAS) in the heart, skeletal muscle, and blood vessels [3,46–48]. Thus, we hypothesize that ET in HF rats reduces tPVAT angiotensin II levels. Figure 4A shows that angiotensin II levels are higher in the tPVAT of uHF than uSO, and surprisingly, refuting our hypothesis, ET did not change this parameter in the tHF animals.

Another critical system involved in PVAT (dys)function and modulated by the effects of ET that could be involved in HF is the noradrenergic system [20,49,50] which is increased in HF [28,51–53]. Thus, we evaluated the tyrosine hydroxylase expression, a key enzyme involved in NE synthesis, and the NE content in the tPVAT of the animals studied. The expression of tyrosine hydroxylase was higher in both HF groups than in uSO (Figure 4B). However, in contrast, NE levels were reduced in uHF compared with uSO, while ET restored them to levels similar to those observed in uSO rats (Figure 4C). Interestingly, this scenario occurred exclusively in tPVAT, since NE plasma levels were higher in uHF rats than uSO, and ET did not modify this parameter in tHF rats (uSO: 2.33 ± 0.24 (*n* = 10) vs. uHF: 5.19 ± 0.77* (*n* = 10) vs. tHF: 5.19 ± 1.23* (*n* = 7) ηg/mL; one-way ANOVA, *P<0.05 vs. uSO).

### Combined exercise training improves the β3-AR/adiponectin/AMPKα1/2 signaling pathway in tPVAT of HF post-MI rats

**Figure 5:**
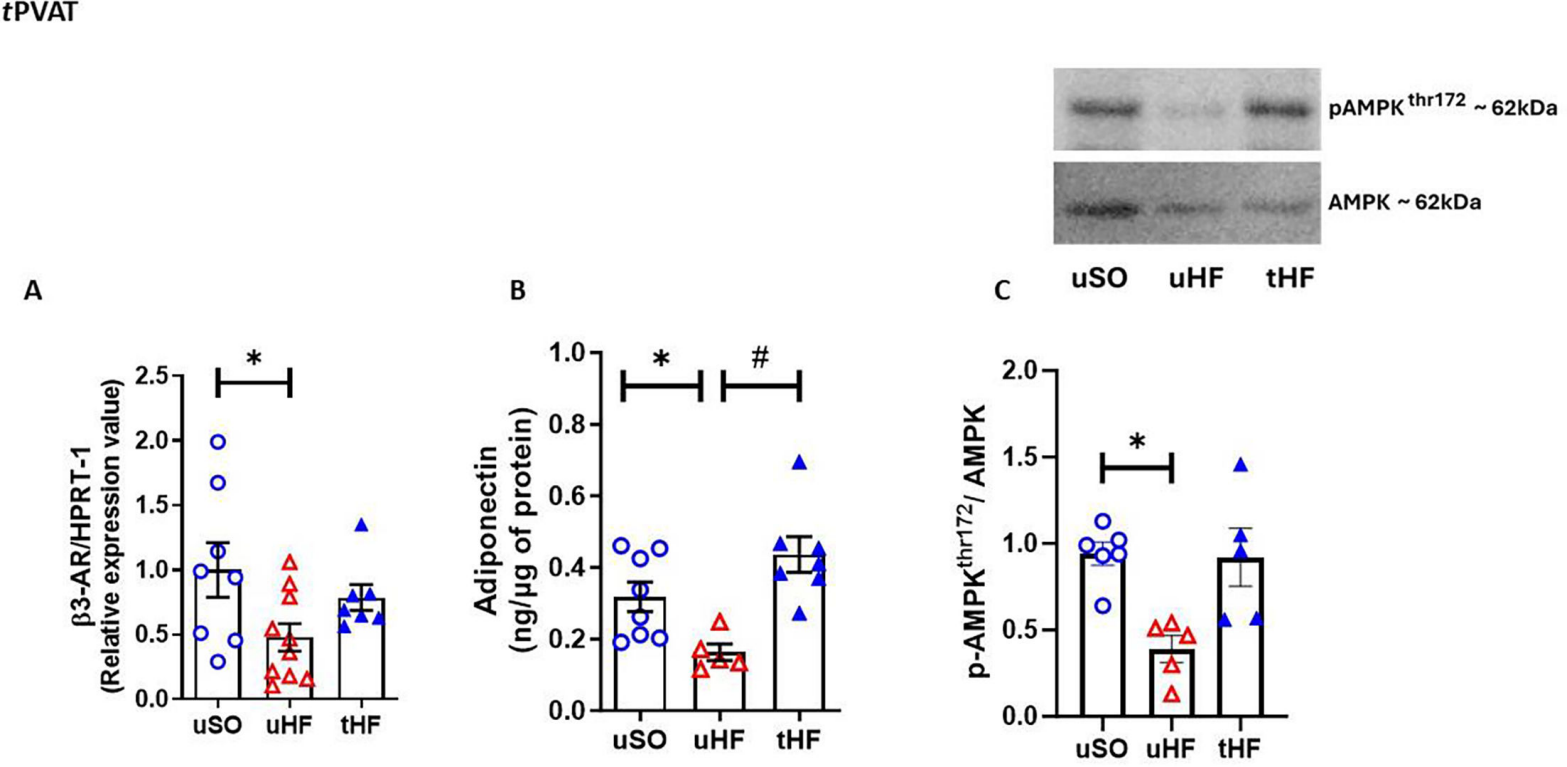
The β3-AR/adiponectin/AMPK signaling pathway is involved in the beneficial effect of exercise training in HF rats. (**A**) Gene expression of the β3-adrenoceptor (AR); (**B**) adiponectin levels; and (**C**) pAMPK^Thr172^ to AMPK ratio protein expression in tPVAT from untrained sham-operated (uSO), untrained heart failure (uHF), and trained heart failure (tHF) rats. All results are expressed as mean ± SEM. Gene expression is presented as the fold of induction to the uSO group. One-way analysis of variance (ANOVA) followed by the Tukey post-hoc test was used to compare the results. *P<0.05 vs. uSO; ^#^ *P*<0.05 vs. uHF.

Next, we evaluated the role of β3-AR/adiponectin/AMPKα1/2 signaling pathway in the beneficial effects of ET on tPVAT anticontractile function. β3-AR gene expression (Figure 5A), adiponectin levels (Figure 5B), and the p^Thr172^AMPK to AMPK protein expression ratio (Figure 5C) were significantly reduced in the tPVAT of the uHF compared with uSO rats. Interestingly, ET enhanced the tPVAT adiponectin levels of tHF rats to the levels observed in uSO rats (Figure 5B), as well as β3-AR gene expression (Figure 5A) and the p^Thr172^AMPK to AMPK protein expression ratio (Figure 5C).

## Discussion

ET has increasingly been recognized as one of the beneficial non-pharmacological interventions widely recommended for treating and preventing cardiovascular diseases [41]. The combination of aerobic exercises and resistance training is included in the guidelines for HF treatment as the ideal recommendation to enhance quality of life and provide additional benefits to patients [34–36]. These benefits extend to the cardiovascular system in cardiovascular diseases [54–56], prompting further exploration of the impact of combined ET on the vasoactive properties of PVAT in an experimental model of HF post-MI. In the present study, we demonstrate for the first time that a combined exercise protocol effectively mitigates tPVAT dysfunction in rats with HF. Subsequently, we investigated the underlying signaling pathways responsible for the beneficial effects of regular ET on tPVAT function.

Our previous findings demonstrated that animals with HF had a significant change in the adipocyte phenotype in tPVAT, changing a thermogenic brown adipocyte to a lipid store profile (whitening), with a reduction in gene expression of brown and beige adipose tissue markers (PRDM-16 and EPSTI-1, respectively) and an increase in the white adipose tissue marker (TCF-21), contributing to the impairment of the anticontractile function of tPVAT found in HF [11]. Our findings align with other studies, which have observed a strong link between HF development and brown adipose tissue dysfunction [57,58]. This relationship was thoroughly investigated using clinical data from patients and various animal models of HF, demonstrating a reduced thermogenic capacity and significant phenotypic changes in brown adipose tissue, as well as its association with HF [58]. Interestingly, in the present study, we found that ET restored the expression of PRDM-16 and EPSTI-1 while reducing the expression of TCF-21, thus inducing browning and reversing the phenotypic changes observed in tPVAT from HF post-MI rats. These data corroborate other studies that have demonstrated the ability of ET to promote browning in PVAT under physiological and cardiometabolic conditions [31,59–61].

The β3-AR signaling pathway stimulates the thermogenic profile of brown adipose tissue in both humans and rodents [62,63]. Additionally, physiological increases in sympathetic noradrenergic stimulation also promote adipose tissue activation through β3-AR [62–64]. β3-AR activates the protein kinase A (PKA) pathway, which induces thermogenic adaptations, leading to lipolysis, mitochondrial biogenesis, and increments in UCP1 expression [63,65]. It is worth noting that, despite the strong link between β3-AR activation and changes in adipose tissue phenotype, the most relevant studies have primarily evaluated different peripheral adipose tissue depots, excluding PVAT. The present results also demonstrate that ET leads to a recovery in UCP1 gene expression in the tPVAT of HF, despite having no significant effect on mitochondrial biogenesis, with mtTFA levels remaining low in the tHF group compared with the uHF group. Thus, our data demonstrate that the association between changes in β3-AR gene expression and adipose tissue markers may also occur in tPVAT of HF rats post-MI and is modulated by ET. We acknowledge, however, that we did not perform experiments to establish the cause-and-effect relationship between β3-AR activation and the expression of brown and beige adipose tissue markers in tPVAT.

The mesenteric PVAT (mPVAT) and tPVAT are known to retain excess NE via reuptake [66], preventing adverse effects such as increased vascular contraction [50]. The functional existence of catecholamines within PVAT, independent of innervation, further highlights their importance for this tissue [50,66]. β3-AR has been detected in tPVAT of rats, mice, and humans [67,68], and its activation is associated with beneficial effects, including the prevention of aortic dissections and aneurysms, as well as the reduction of PVAT inflammation [68]. It is also well established that NE induces β3-AR activation, promoting adiponectin release and the anticontractile effect of mPVAT [50,64]. Our data demonstrated that β3-AR gene expression is lower in the tPVAT of uHF rats than in uSO, suggesting an impaired signaling pathway in HF. This is supported by two key findings: tyrosine hydroxylase, a pivotal limiting step in NE synthesis, showed higher expression in uHF, yet NE content in tPVAT was reduced. This dysfunction is compounded by the whitening of tPVAT, as discussed above, and lower adiponectin secretion in uHF compared with uSO, ultimately leading to the loss of tPVAT anticontractile function. Interestingly, ET was effective in inducing browning and restoring adiponectin levels, NE content, and β3-AR gene expression in the tPVAT. Similar data were previously published in the mPVAT of obese mice, showing downregulation of the β3-AR/adiponectin signaling pathway, which was restored by aerobic training [20,50].

Although the exact mechanism by which adiponectin contributes to the anticontractile effects of PVAT is not fully understood, the presence of the AdipoR1 receptor in PVAT (such as tPVAT and mPVAT) [69,70], as well as in the vascular endothelium and smooth muscle, is noteworthy [33,71]. The activation of AdipoR1 is linked to the stimulation of AMPK in various tissues, including cardiac, skeletal, and vascular muscle [72,73]. Consequently, the literature suggests that the signaling pathway by which adiponectin promotes the anticontractile effect of PVAT involves its binding to the AdipoR1 receptor, which stimulates AMPK/eNOS activation [33,70,74]. Remarkably, our results demonstrate that the NE/β3-AR/adiponectin/p^Thr172^AMPK/eNOS pathway is impaired in tPVAT of HF rats post-MI. This impairment is associated with a reduced L-NAME effect on phenylephrine-induced contraction in PVAT+ aortic rings. Conversely, combined training effectively restored this adiponectin pathway to SO levels and enhanced the L-NAME effect in aortic rings, thereby improving the anticontractile effect of tPVAT in HF rats.

Interestingly, our data shows a differential impact of ET on local (tPVAT) and systemic NE levels. Once exercise restores the lower tPVAT NE content of HF rats toward SO levels, it does not alter the elevated systemic NE available in HF rats. This observation suggests that the beneficial effects of training may include a local modulation of NE transport and/or metabolism in the PVAT. The NE reuptake by PVAT involves plasma membrane transporters, such as the organic cation transporter 3 and norepinephrine transporter, followed by vesicular deposition via vesicular monoamine transporter (VMAT)-1 and VMAT-2 [50,66,75,76]. Furthermore, NE metabolism, primarily through semicarbazide-sensitive amine oxidase and, to a lesser extent, by monoamine oxidase A, has been described as a key system for reducing the availability of this catecholamine [75]. Although these NE handling mechanisms are crucial for the pathways investigated in the present study, experimental constraints, combined with the low basal gene expression of these specific targets in PVAT [66,75], prevent us from determining whether HF and ET influence reuptake and/or catecholamine metabolism pathways in tPVAT.

PVAT is a type of connective tissue comprising various cell types. The majority of these cells are adipocytes, but they also contain mesenchymal stem cells, pre-adipocytes, fibroblasts, nerves, endothelial cells, and various immune cells, including macrophages, lymphocytes, and eosinophils [77]. In this way, increases in pro-inflammatory and pro-oxidative markers in PVAT, which contribute to its dysfunction, have been described in several cardiovascular diseases [11,15,19–21,78,79]. Low-grade inflammation and oxidative stress also occur in the heart, vessels, and systemically during HF [11,80]. Our previous study characterized oxidative stress in the tPVAT of HF rats post-MI and a reduced anticontractile effect of tPVAT [11]. In the present study, we reinforce these findings with new data showing that the tPVAT of HF rats also exhibits a pro-inflammatory phenotype. In addition, combined ET effectively reverses both phenotypes, restoring oxidative status, reducing pro-inflammatory cytokines (IL-6, TNF-α, and MCP-1), and increasing anti-inflammatory cytokines (IL-10). These phenotypic changes promoted by combined exercise were accompanied by increased NO bioavailability in the tPVAT. Extrapolating to other experimental models of cardiometabolic disorders, several studies using an animal model of obesity have also found that long-term aerobic exercise protocols have a beneficial impact on the antioxidant and anti-inflammatory status of tPVAT [20,31,32,81]. However, our results represent the first demonstration that combined ET effectively restores oxidative and inflammatory signaling pathways in tPVAT from HF post-MI rats.

As previously described by our research group and other researchers, angiotensin II is associated with a pro-oxidant and pro-inflammatory profile in the cardiovascular system, including PVAT [11,15]. In addition, our previous findings demonstrated an association between RAS hyperactivation and tPVAT dysfunction in HF rats [11]. Therefore, we also hypothesize that combined ET mitigates this RAS hyperactivation in tPVAT. It is essential to emphasize that RAS components are physiologically released by tPVAT, acting as vasoconstrictor or vasodilator factors, which contribute to the maintenance of vascular tone; however, the imbalance of these factors is associated with PVAT dysfunction [11,82–84]. In the present study, we observed detrimental effects of tPVAT, possibly caused by RAS hyperactivation, which include alterations in oxidative status, NO bioavailability, and chronic inflammation. Moreover, refuting our hypothesis, angiotensin II levels remained at higher concentrations in the tPVAT of tHF rats than those observed in uSO rats. Likewise, these data lead us to two hypotheses that merit further investigation: (1) that the effects of combined aerobic and resistance exercise may not have been able to mitigate the local RAS hyperactivation caused by HF, in a way that the improvements seen in the tHF group may be entirely independent of the local RAS, and the elevated angiotensin II levels in the tPVAT may be limiting the ability of the combined ET protocol to fully restore the anticontractile properties; or (2) that the combined aerobic and resistance ET altered systemic RAS hyperactivation, as this is well described in the literature [3,85]—although our assessment, restricted to tPVAT, was not able to detect this change.

It is important to know that a limitation of our study is the exclusion of female animals. While recognizing the importance of researching both sexes in HF, our decision was based on existing studies that demonstrate significant differences in HF progression and signs between males and females. Previous research indicates that female rats experience milder hemodynamic and morphometric changes, as well as fewer HF signs, post-MI than males [86,87]. This point is crucial for our study, as HF severity significantly affects vascular reactivity, especially with permanent coronary artery occlusion [6]. Thus, we focus the present study on male rats to minimize variability and enhance the accuracy of our findings. However, future research should focus on female responses to evaluate and deepen their specificities.

**Figure 6:**
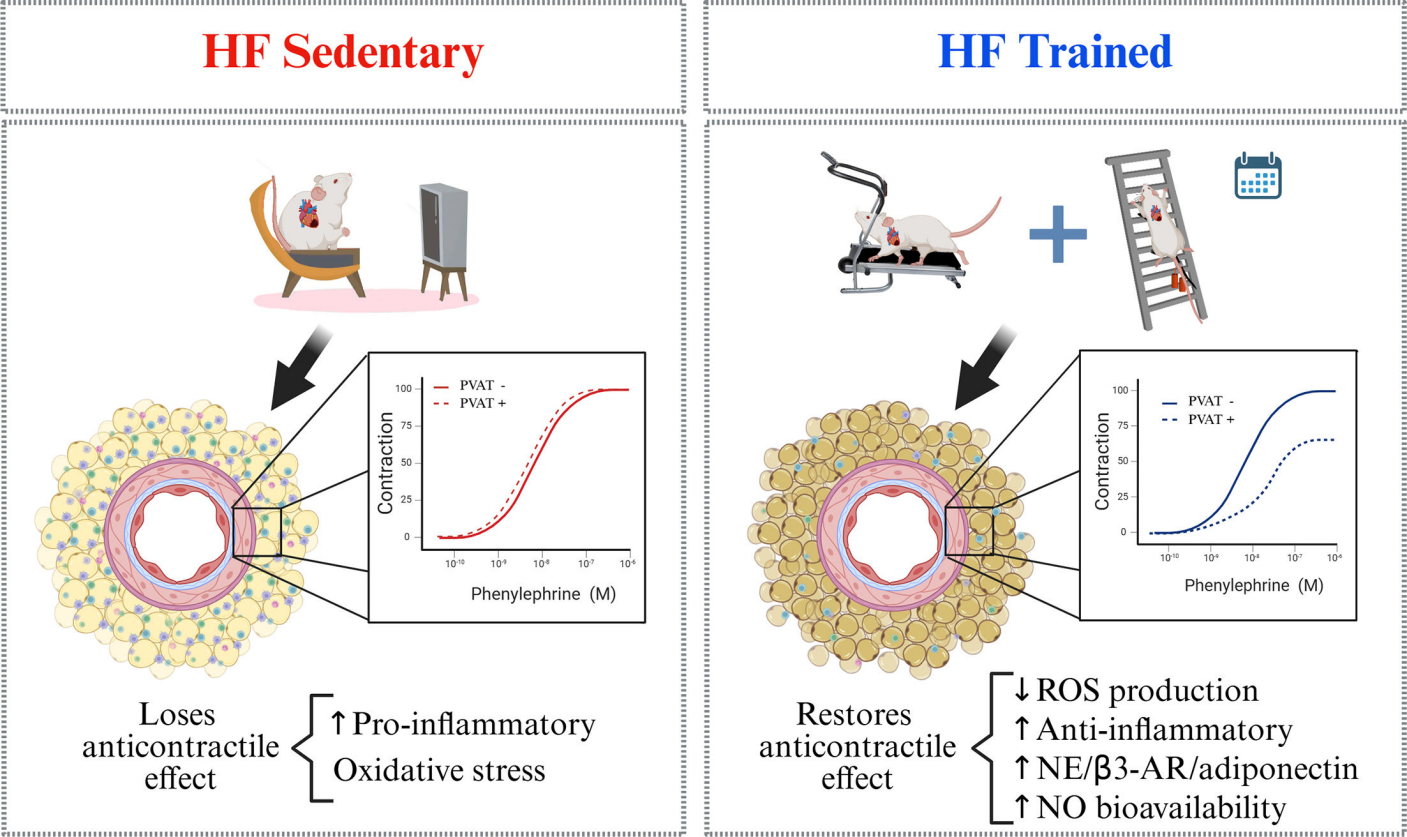
Main adjustments found in the tPVAT of untrained and exercise-trained heart failure rats. The image summarizes the main findings observed in tPVAT of untrained (sedentary) and 8-week combined aerobic and resistance-trained heart failure (HF) rats. These findings highlight the potential of exercise training as a non-pharmacological approach to managing PVAT and vascular adjustments in HF. Image created with BioRender.com.

Taken together, this study’s main findings demonstrate that combined aerobic and resistance ET can restore the protective anticontractile effect of tPVAT in HF post-MI by promoting adipocyte browning, improving the antioxidant and anti-inflammatory status, and enhancing the NE/β3-AR/adiponectin/AMPK/eNOS/NO pathway (Figure 6). Our findings are the first to emphasize the beneficial impact of a combined exercise protocol on the vasoactive properties of tPVAT in HF, highlighting the importance of ET as a first-line treatment for vascular complications in HF.

**Clinical Perspectives**

- Exercise training (ET) is widely recognized as a non-pharmacological strategy to improve cardiovascular health, prompting an investigation into its specific effects on the vasoactive properties of thoracic perivascular adipose tissue (tPVAT) in heart failure (HF) post-myocardial infarction.
- This study demonstrates that combined aerobic and resistance training reverses HF-induced tPVAT dysfunction by restoring brown adipocyte phenotype, reducing inflammation and oxidative stress, and reactivating the noradrenaline/β3-adrenoceptor/adiponectin/AMP-activated protein kinase/endothelial nitric oxide synthase signaling pathway.
- These findings suggest that structured ET may be a potent therapeutic intervention to restore PVAT function and improve vascular health in HF patients, beyond its systemic cardiovascular benefits.

## Supplementary material

online supplementary material 1

## Data Availability

The data supporting this study’s findings are available from the corresponding author upon request. The uncropped and unedited versions of the Western blot figures are presented as a supplementary file.

## Open Access

This article has been published open access under our Subscribe to Open programme, made possible through the support of our subscribing institutions, learn more here: https://portlandpress.com/pages/open_access_options_and_prices#conditional

## Abbreviations

AMPK: AMP-activated protein kinase
ANOVA: analysis of variance
AR: adrenoceptor
BNP: brain natriuretic peptide
DHE: dihydroethidium
EPSTI-1: epithelial stromal interaction 1
ET: exercise training
HF: heart failure
IL-6: interleukin-6
IL-10: interleukin-10
LV: left ventricle
LVSP: LV systolic pressure
LVeDP: LV end-diastolic pressure
MCP-1: monocyte chemoattractant protein-1
MI: myocardial infarction
NE: noradrenaline
NO: nitric oxide
PKA: Protein Kinase A
PRDM-16: PR-domain containing 16
PVAT: perivascular adipose tissue
PVDF: polyvinylidene fluoride
RAS: renin-angiotensin system
ROS: reactive oxygen species
RV: right ventricle
SO: sham operation
TCF-21: transcription factor 21
TNF-α: tumor necrosis factor-α
UCP1: uncoupling protein 1
VMAT: vesicular monoamine transporter
eNOS: endothelial nitric oxide synthase
mtTFA: mitochondrial transcription factor A
tHF: trained HF
tPVAT: thoracic perivascular adipose tissue
tSO: trained SO
uHF: untrained HF
uSO: untrained SO
