# Supplementary material for: Combined aerobic and resistance exercise training restores perivascular adipose tissue function in the thoracic aorta of rats with heart failure
Source: Clin Sci (Lond). 2025 Nov 12;139(21):1355–71. doi: 10.1042/CS20256965 (PMC12751056; doi:10.1042/CS20256965)

Supplementary material: Full uncropped and unedited versions of the Western-blots figures

**Exercise training ameliorates perivascular adipose tissue function in the thoracic aorta of heart failure rats.**

Milene Tavares Fontes^1^, Daniela Esteves Ferreira dos Reis Costa^1^, Patrizia Dardi^1^, Suliana Mesquita Paula^1^, Gisele Kruger Couto^1^, Érique de Castro^1^, Luciana Venturini Rossoni^1^

^1^Department of Physiology and Biophysics, Institute of Biomedical Sciences, University of Sao Paulo, Sao Paulo, Brazil.

**Corresponding author:** Luciana Venturini Rossoni

**E-mail address:** lrossoni@icb.usp.br

**Postal address:** Instituto de Ciências Biomédicas – ICB I, Universidade de São Paulo, Av. Prof. Lineu Prestes, 1524 – Sala 225, Cidade Universitária – Butantã, São Paulo, SP – Brasil, CEP: 05508-000

**Supplementary Figure 1: Thoracic aorta perivascular adipose tissue (tPVAT) - uncropped and unedited versions of the Western blots** **figure presented in manuscript Figure 4B.** Total protein expression of tyrosine hydroxylase (TH) and its respective ponceau staining in tPVAT from untrained SHAM-operated (uSO), untrained heart failure (uHF), and training heart failure (tHF) rats.


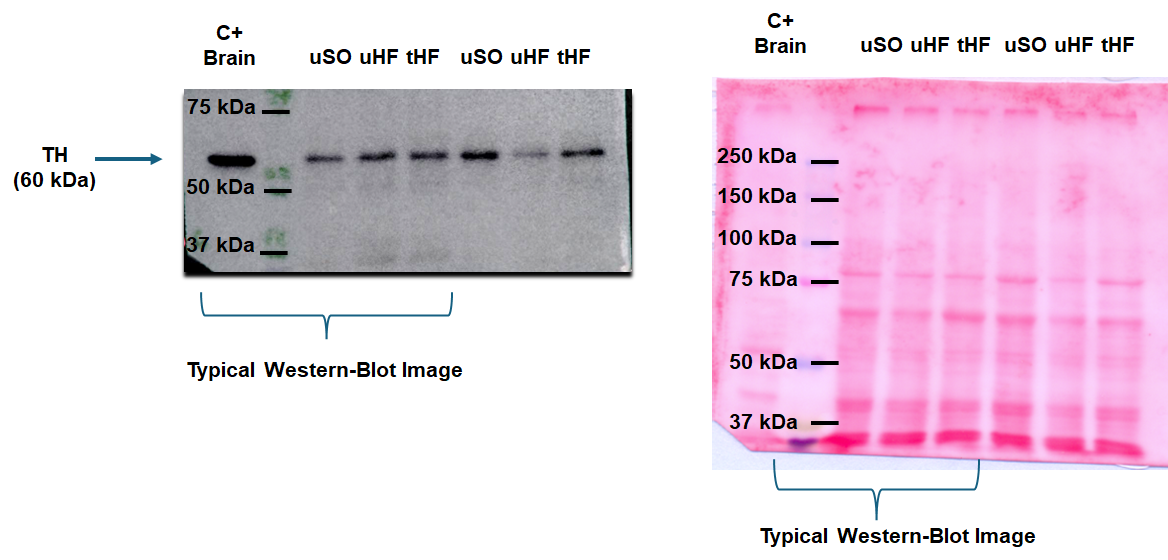


**Supplementary Figure 2: Thoracic aorta perivascular adipose tissue (tPVAT) - uncropped and unedited versions of the Western blots figure presented in manuscript Figure 5C.** The ratio of pAMPK^Thr172^ to total AMPK protein expression in tPVAT from untrained SHAM-operated (uSO), untrained heart failure (uHF), and training heart failure (tHF) rats.


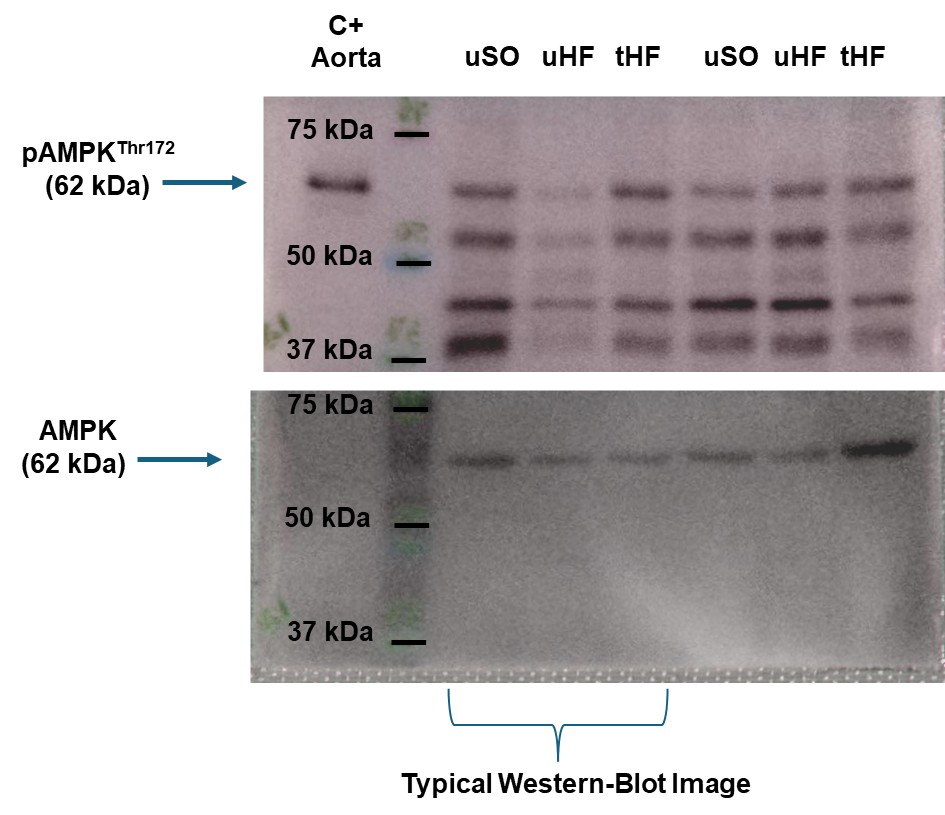

Supplement: online supplementary material 1 [file CS-139-21-CS20256965-s001.docx]
